# CMR-based blood oximetry via multi-parametric estimation using multiple T2 measurements

**DOI:** 10.1186/s12968-017-0403-1

**Published:** 2017-11-09

**Authors:** Juliet Varghese, Lee C. Potter, Richard LaFountain, Xueliang Pan, Subha V. Raman, Rizwan Ahmad, Orlando P. Simonetti

**Affiliations:** 1 Dorothy M. Davis Heart and Lung Research Institute, The Ohio State University Wexner Medical Center, Columbus, OH USA; 2 Department of Electrical and Computer Engineering, The Ohio State University, Columbus, OH USA; 3 Department of Health and Exercise Science, The Ohio State University, Columbus, OH USA; 4 Center for Biostatistics, The Ohio State University, Columbus, OH USA; 5 Division of Cardiovascular Medicine, Department of Internal Medicine, The Ohio State University Wexner Medical Center, Columbus, OH USA; 6 Department of Biomedical Engineering, The Ohio State University, Columbus, OH USA; 7 Department of Radiology, The Ohio State University Wexner Medical Center, Columbus, OH USA

**Keywords:** Oxygen saturation, T2 mapping, Cardiovascular magnetic resonance

## Abstract

**Background:**

Measurement of blood oxygen saturation (O2 saturation) is of great importance for evaluation of patients with many cardiovascular diseases, but currently there are no established non-invasive methods to measure blood O2 saturation in the heart. While T2-based CMR oximetry methods have been previously described, these approaches rely on technique-specific calibration factors that may not generalize across patient populations and are impractical to obtain in individual patients. We present a solution that utilizes multiple T2 measurements made using different inter-echo pulse spacings. These data are jointly processed to estimate all unknown parameters, including O2 saturation, in the Luz-Meiboom (L-M) model. We evaluated the accuracy of the proposed method against invasive catheterization in a porcine hypoxemia model.

**Methods:**

Sufficient data diversity to estimate the various unknown parameters of the L-M model, including O2 saturation, was achieved by acquiring four T2 maps, each at a different *τ*_180_ (12, 15, 20, and 25 ms). Venous and arterial blood T2 values from these maps, together with hematocrit and arterial O2 saturation, were jointly processed to derive estimates for venous O2 saturation and other nuisance parameters in the L-M model. The technique was validated by a progressive graded hypoxemia experiment in seven pigs. CMR estimates of O2 saturation in the right ventricle were compared against a reference O2 saturation obtained by invasive catheterization from the right atrium in each pig, at each hypoxemia stage. O2 saturation derived from the proposed technique was also compared against the previously described method of applying a global calibration factor (*K*) to the simplified L-M model.

**Results:**

Venous O2 saturation results obtained using the proposed CMR oximetry method exhibited better agreement (y = 0.84× + 12.29, R^2^ = 0.89) with invasive blood gas analysis when compared to O2 saturation estimated by a global calibration method (y = 0.69× + 27.52, R^2^ = 0.73).

**Conclusions:**

We have demonstrated a novel, non-invasive method to estimate O2 saturation using quantitative T2 mapping. This technique may provide a valuable addition to the diagnostic utility of CMR in patients with congenital heart disease, heart failure, and pulmonary hypertension.

## Background

Blood oxygen saturation (O2 saturation) is a relevant biomarker in many cardiovascular diseases; O2 saturation is used to determine the presence and severity of intra- and extra-cardiac shunts in congenital heart disease, and to provide an index of systemic oxygen delivery and consumption in heart failure and pulmonary artery hypertension [1, 2]. Invasive cardiac catheterization is required to measure O2 saturation within the cardiac chambers, the pulmonary arteries, the vena cavae, and other deep vessels; however, this procedure is expensive, invasive, and carries associated risks [3, 4]. A non-invasive alternative to measure O2 saturation is, therefore, highly desired.

Based on the magnetic properties of oxygenated (diamagnetic) and deoxygenated (paramagnetic) hemoglobin in blood, non-invasive magnetic resonance (MR) techniques have been previously described to estimate blood O2 saturation, myocardial and skeletal tissue oxygenation, and brain oxygen extraction fraction [5–8]. These methods exploit the dependence of MR relaxation times (T1, T2 and T2*) on the oxygen saturation of hemoglobin in blood [5, 9, 10].

The transverse relaxivity (R2 or 1/T2) of blood may be influenced by a number of physiological, dynamic, and pulse sequence dependent factors. The Luz-Meiboom (L-M) chemical exchange model [11] has been used to mathematically characterize the relation between T2 of whole blood and its O2 saturation. The model originally describes the transverse decay that results from the transfer of protons between a protein and a water solution, and the dependence of the measured spin echo decay on the inter-echo spacing of a Carr-Purcell Meiboom Gill (CPMG) sequence. It has since been applied to define the apparent 1/T2 of whole blood as the sum of the inherent and proton exchange-dependent relaxation rates of red blood cells (RBC) which contain the hemoglobin that binds oxygen, and plasma, when measured by a CPMG refocusing pulse train or by means of a T2 weighted magnetization prepared sequence.

Efforts to accurately characterize this relationship between T2 and O2 saturation of whole blood have led to experimental and theoretical parameterization of the L-M model with varying degrees of model complexity, including segregating the contributions from individual paramagnetic and diamagnetic blood components [5, 12, 13]. Wright et al. [5] was the first to quantitatively estimate blood oxygen saturation from an in vivo measurement of blood T2. They described the L-M model as

1 $$ \frac{1\ }{T_{2b}}=\frac{1}{T_{2O}}+(Pa)\left(1- Pa\right){\tau}_{ex}{\left[\left(1-\frac{\%{SbO}_2}{100}\right)\alpha {\omega}_0\right]}^2\times \left(1-\frac{2{\tau}_{ex}}{\tau_{180}}\mathit{\tanh}\frac{\tau_{180}}{2{\tau}_{ex}}\right), $$

where *T*_2*b*_ is the T2 relaxation time of blood (arterial or venous), *T*_2*O*_ is the T2 of fully oxygenated blood, *Pa* is the fraction of protons at one of the two sites undergoing chemical exchange, *τ*_*ex*_ is the water proton exchange time between erythrocytes and plasma, *α* is a dimensionless parameter that is dependent on the susceptibility difference of deoxy- and oxyhemoglobin and the geometry of the red blood cells, *ω*_0_ is the proton resonance frequency (fixed for a given static field strength), %*SbO*_2_ is the oxygen saturation (arterial or venous), and *τ*_180_ is the inter-echo spacing of the 180° refocusing pulses in the CPMG echo train or the T2 preparation module. The term $\alpha {\omega}_0\left(1-\frac{\%{SbO}_2}{100}\right)$ represents the frequency difference between the protons in erythrocytes and plasma. *Pa* has been described as 0.9 times or equal to hematocrit (*Hct*) [13, 14], the volume fraction of RBC in whole blood. We assume *Pa* to be equal to *Hct* in our implementation of the L-M model.

In this model, there are several unknown biophysical parameters besides O2 saturation that are experimentally difficult to determine. In vitro studies have been performed using static or flowing human or animal blood in order to determine these unknown parameters; however, the accuracy of estimation is prone to variability in experimental conditions [15]. It is also unclear whether these biophysical parameters may be different under dynamic in vivo conditions, across species, or between healthy and diseased individuals. Nevertheless, previous MRI-based approaches to determine O2 saturation from T2 measurements have typically relied on either assigning some reasonable values to these unknown model parameters from published literature [6, 16–19] or performing separate in vitro calibration [12, 20–23] to estimate the parameter values.

These parameters are typically calibrated for specific imaging conditions, such as for a particular inter-echo spacing or field strength. A commonly used simplified model, initially proposed by Wright et al. [5] is shown in Eq. 2:

2 $$ \frac{1}{T_{2b}}=\frac{1}{T_{2O}}+K{\left[\left(1-\frac{\%{SbO}_2}{100}\right)\right]}^2, $$

where *T*_2*b*_ is the measured T2 value of blood; *T*_2*O*_ is the T2 value of fully oxygenated blood, which is either assumed or measured in vitro; *K* is a calibration factor derived from in vitro experiments and incorporates parameters such as *Hct*, *τ*_*ex*_, *α*, and *τ*_180_; and %*SbO*_2_ is the parameter of interest, i.e. the blood O2 saturation. While the use of a simplified model with a fixed calibration factor may offer greater computational ease, it comes at the cost of reduced accuracy and precision. As an example, due to the dependence of *K* on hematocrit, the calibrated factor from an in vitro calibration process performed over a normal range of hematocrit may not be accurate in anemic or polycythemic patients [24].

To overcome the inaccuracies of a globally calibrated model, patient-specific calibration has also been proposed [5]. However, this process requires drawing a significant volume of blood, in vitro oxygenation and imaging of multiple blood samples for each individual patient [18, 23]. The processes of image acquisition, calibration, and off-line processing are time-consuming and have hindered widespread clinical application of previously described oximetry techniques.

Therefore, for successful clinical application in the evaluation of cardiovascular disease, T2-based cardiovascular MR (CMR) oximetry must (i) reliably and accurately estimate blood T2 across various cardiac and vascular locations, (ii) provide a patient-specific estimate of O2 saturation by accounting for inter-individual variability of the other biophysical parameters in the L-M model, and (iii) utilize a clinically practical procedure that entails efficient data acquisition and analysis. Taking these factors into consideration, we propose a framework for patient-specific, T2-based CMR oximetry. We hypothesize that a more accurate estimation of O2 saturation is obtained by solving the L-M model using multiple T2 maps. The estimation of O2 saturation and other nuisance parameters in the L-M model is posed as a non-linear least squares (NLLS) problem, with constraints enforced on the nuisance parameters. We validated this technique against invasive blood gas analysis in a porcine model of graded hypoxemia.

### Theory

In the model described in Eq. 1, *T*_2*b*_ can be measured using CMR, and *Hct* (*Pa*) can be measured from a small blood sample. The parameters *ω*_0_ and *τ*_180_ can be controlled based on the choice of magnetic field strength and T2-preparation pulse design, respectively. This leaves the desired blood oxygen saturation, %*SbO*_2_, and three other nuisance parameters (*T*_2*O*_, *τ*_*ex*_, and *α*) as unknowns.

**Fig. 1:**
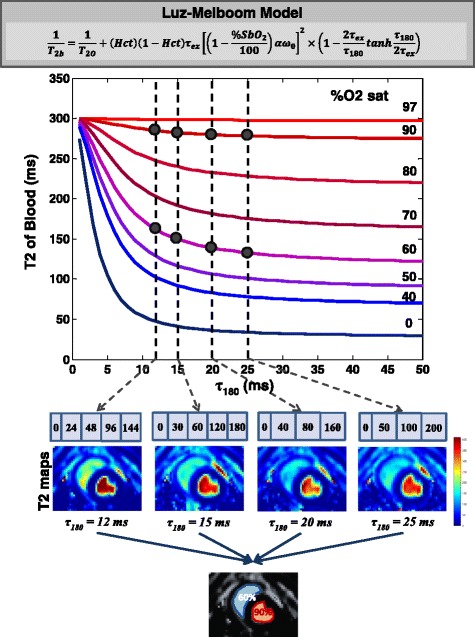
Overall framework of proposed CMR oximetry technique. The graph depicts the simulation of the L-M model (described in gray box at top of the figure, adapted from [14]). The transverse relaxation time of blood is plotted as a function of O2 saturation ( %*SbO*_2_) and inter-echo spacing (*τ*_180_). Other parameters used in the simulation were: *Hct* = 0.41, *T*_2*O*_ = 300 ms, *α* = 0.545 ppm, and *τ*_*ex*_ = 3 ms. Four different T2 maps, each with a fixed *τ*_180_ at 12, 15, 20, and 25 ms, were acquired to generate a set of equations for the L-M model relating T2 and O2 saturation. The data, along with known *Hct* and arterial O2 saturation, are jointly processed by NLLS curve fitting to estimate the unknown parameters - venous O2 saturation, *α*, *τ*_*ex*_, and *T*_2*O*_

While a single measurement of the apparent blood T2 would not be sufficient to estimate all of the unknown patient-specific parameters in the L-M model, we propose here a method that allows these unknown parameters to be estimated, within a range, on a patient-specific basis. Noting that *τ*_180_ is a controllable parameter, acquiring multiple T2 measurements, each at a different *τ*_180_, provides the diversity of data that is needed to characterize the patient-specific relationship between blood T2 and O2 saturation. Once a sufficient number of T2 values have been acquired, each using a different *τ*_180_, the four unknown model parameters (%*SbO*_2_, *T*_2*O*_, *τ*_*ex*_, and *α*) can be estimated using a NLLS curve fit. Under high signal-to-noise (SNR) conditions, the solution remains viable when the nuisance parameters are unknown a priori*.* However, in order to discourage convergence to local minima and to combat loss of estimation sensitivity in the presence of noise, we opted to constrain the nuisance parameters. This approach provides a framework for patient-specific T2 oximetry (shown in Fig. 1), and eliminates reliance on generic and potentially inaccurate calibration factors [25, 26].

Our lab has previously developed a technique to perform rapid, quantitative characterization of the myocardial T2 to identify inflammation and edema [27, 28]. The T2 mapping sequence consists of the acquisition of several single-shot T2 weighted images at specific T2 preparation times. Each of these images are acquired using a non-selective T2 preparation module, consisting of a 90^0^ tip-down RF pulse, a train of Malcolm Levitt (MLEV) phase cycled composite 180^0^ (90^0^–180^0^–90^0^) refocusing RF pulses, ending with a composite 90^0^ (270^0^–360^0^) tip-up RF pulse. The T2 preparation is immediately followed by a balanced steady-state free precession (bSSFP) readout, with linear traversal across k-space data. The T2 mapping sequence also employs a non-rigid registration motion correction algorithm to correct for motion occurring between the different T2-prepared images. The signal in each of the motion corrected T2 prepared images are fit pixel-wise to a mono-exponential decay curve as a function of T2p, resulting in a quantitative T2 map of the region being imaged.

**Fig. 2:**
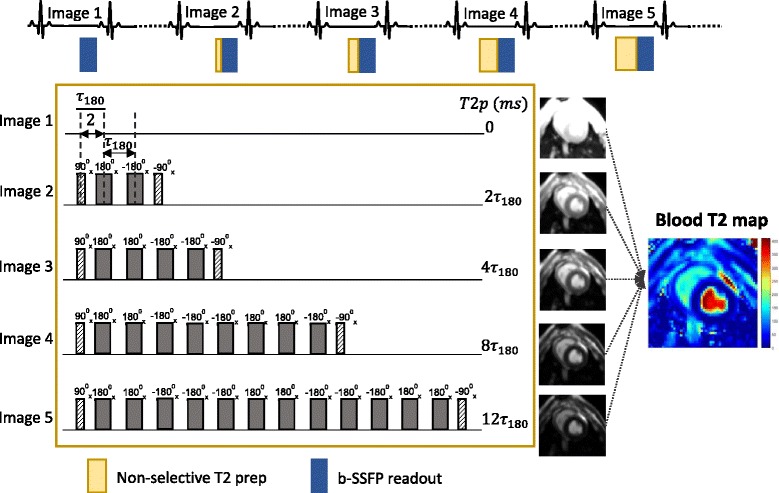
Pulse sequence scheme for acquisition of quantitative blood T2 map. The figure depicts the scheme for the T2 preparation module to acquire a single quantitative T2 map of the blood for a given *τ*_180_. Four such T2 maps were acquired at different *τ*_180_ for the estimation of blood O2 saturation

In this technique, which was primarily designed for myocardial T2 measurement, *τ*_180_ was adjusted among a fixed number of refocusing pulses in the T2 preparation train in order to vary the echo times, T2p, which is defined as T2p = number of refocusing pulses x *τ*_180_. For the estimation of blood O2 saturation, we extend T2p by increasing the number of refocusing pulses based on a segmented Malcolm Levitt phase cycling pattern (0, 2, 4, 8, and 12 refocusing pulses) [29]. Thus, we generate four T2 maps for a given blood pool, using *τ*_180_ values of 12, 15, 20, and 25 ms. The T2p times corresponding to each T2 map used in the study were therefore 0, 24, 48, 96, and 144 ms (for *τ*_180_ = 12 ms); 0, 30, 60, 90, and 180 ms (for *τ*_180_ = 15 ms); 0, 40, 80, and 160 ms (for *τ*_180_ = 20 ms); and 0, 50, 100, and 200 ms (for *τ*_180_ = 25 ms), respectively. The number of T2p images used to generate each map was chosen such that the latest T2p for each T2 map remained within a close range, around the expected T2 values of arterial and venous blood. An illustration of the T2 mapping scheme is shown in Fig. 2.

As the CMR imaging planes of the heart usually include both arterial and venous blood pools, each T2 map can provide a T2 measurement of both arterial and venous blood. It is also possible to measure the O2 saturation of arterial blood by non-invasive pulse oximetry. Therefore, joint processing of venous and arterial blood T2 measurements, together with a known value of arterial O2 saturation, can provide additional information that aids in accurate parameter estimation.

In summary, to derive an estimate of blood O2 saturation from its corresponding T2, we acquired four T2 maps resulting in eight equations: four for the venous blood of interest and four for a reference arterial blood pool. We propose that these T2 measurements, together with the hematocrit and non-invasive arterial O2 saturation, collectively support a reliable fit of the L-M model. As shown in Fig. 1, our solution for non-invasive, in vivo estimation of O2 saturation thus involves a two-step procedure: (i) acquire distinct, quantitative blood T2 maps, each at a specific *τ*_180_, and (ii) utilize a NLLS fitting approach to jointly estimate venous O2 saturation along with the remaining unknown nuisance parameters of the L-M model.

## Methods

### Hypoxemia protocol

**Fig. 3:**
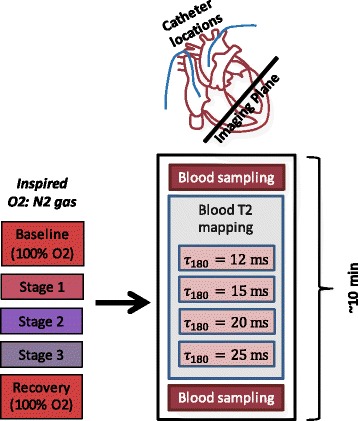
Setup for animal hypoxemia experiment. Each animal was allowed to inspire different concentrations of oxygen (*FiO*_2_). At each level of *FiO*_2_, arterial and venous blood O2 saturation were measured by invasive catheter sampling and blood gas analysis before and after the acquisition of CMR data. Blood T2 was measured in the ventricles by means of T2 maps. Blood T2 measured in the right and left ventricles, along with hematocrit and arterial O2 saturation, were then analyzed for each data set to determine venous O2 sat in the right ventricle

The study was conducted with the approval of the Institutional Animal Care and Use Committee. Seven pigs were anesthetized with isoflurane and mechanically ventilated on 100% oxygen. Balloon catheters were inserted into the right atrium and proximal aorta for sampling venous and arterial blood, respectively. After placement in the CMR scanner, the animals were subjected to controlled graded hypoxemia by varying the ratio of oxygen to nitrogen gas inhaled [30]. We sought to achieve arterial O2 saturation levels ranging from 100% down to 70% in each animal. The experimental set up is illustrated in Fig. 3.

Each inspired gas mixture was maintained for at least 10 min to allow O2 saturation levels to stabilize before blood sampling and imaging. Arterial and venous blood samples (roughly 0.1 mL) were drawn from aortic and right atrial catheters before and after imaging at each hypoxemic stage; the samples were immediately analyzed with a Vetscan I-stat 1 handheld blood gas analyzer (Abaxis Inc., Union City, California, USA). The arterial and venous O2 saturation for each hypoxemic stage was determined by averaging the saturation levels measured before and after imaging (approximately ten minutes apart). The hematocrit was measured in each blood sample and averaged across all measurements to determine the value for each animal.

After stepping through stages from highest to lowest level of inspired oxygen, the animal was allowed to recover for approximately 15–20 min by breathing 100% oxygen. The animal was then euthanized after a second set of measurements was made at 100% arterial O2 saturation. The two sets of baseline blood gas measurements obtained from each animal before and after CMR imaging were tested to evaluate the reproducibility of O2 saturation measurements by catheterization and blood gas analysis.

### CMR protocol

All imaging was performed on a 1.5 T magnet (MAGNETOM Avanto, Siemens Healthineers, Erlangen, Germany) with a maximum gradient amplitude of 45 mT/m and slew rate of 200 mT/m/ms. A flexible six-element phased array body coil was placed on the thorax over the heart and combined with elements of a spine array coil for signal reception. CMR at each stage of hypoxemia included the acquisition of four T2 maps, each using a different *τ*_180_, in a single short axis view including both right and left ventricles. At each stage of hypoxemia, four T2 maps, each with *τ*_180_ of 12, 15, 20, and 25 ms, were acquired in a randomized order to avoid any bias that may be caused by the drifting of the O2 saturation levels. The images were cardiac triggered and acquired free breathing in late systole to avoid rapid, disrupted flow during diastolic filling. The imaging parameters were: TR (time between T2 prepared image acquisitions) 4000 to 5000 ms (seven to fourteen cardiac cycles depending on heart rate, chosen to ensure adequate T1 recovery), two signal averages (NEX), where each NEX is a single shot measurement, flip angle = 70^0^, parallel acceleration = 2, bandwidth = 1182 Hz/pixel, field of view = 400 × 368 mm, matrix size = 128 × 118, spatial resolution = 3.1 × 3.1 × 10 mm. The TE of the SSFP readout was 0.89 ms while TR was 2.2 ms. Parallel acceleration was used in combination with multiple NEX to keep the shot time short while preserving SNR. The acquisition time of each map was approximately 40 to 50 s. Cardiac output was measured at the aortic outflow using a real-time velocity sequence [31] at each hypoxemia stage (TR/TE = 96.4/5.1 ms, TA = 5 s, spatial resolution = 3.8 × 3.1 × 10 mm). Heart rate was monitored using a 3-lead wireless electrocardiogram, and arterial O2 saturation was monitored by placing a pulse oximeter probe on the lower lip of the animal.

### Image analysis

Image analysis was performed on a Leonardo workstation (Siemens Healthineers, Erlangen, Germany). Prior to performing the animal study, the four T2 maps were evaluated on static phantoms prepared to approximate T2 value of arterial and venous blood. The T2 values of the phantoms were measured by placing a region of interest in each of the four maps. These values were compared against a reference T2, measured by a multi-echo turbo spin echo (MESE) sequence. The reference T2 values were calculated by mono-exponential fitting of the signal to the echo times in the MESE sequence. For the animal experiment, the T2 values of arterial and venous blood were measured by manually drawing contours around the lumen of the right ventricular (venous) and left ventricular (arterial) blood pools in each of the four T2 maps acquired at each hypoxemia stage.

For the proposed method, parameter estimation of the L-M model (Eq. 1) was performed using a NLLS curve fit based on an interior point algorithm in Matlab R2016a (Mathworks, Natick, Massachusetts, USA). Blood T2, hematocrit, and arterial O2 saturation (by blood gas analysis) were all measured, and *ω*_0_ (4 × 10^8^ rad/s at 1.5 T) and *τ*_180_ (12, 15, 20, and 25 ms) were known. The eight measurements of blood T2 (four arterial and four venous from the four T2 maps), along with the measured hematocrit and reference arterial O2 saturation at each stage were processed jointly to estimate the O2 saturation in the right ventricular cavity.

For the animal experiment, we tested and compared results from four different approaches. Approach 1: Estimate O2 saturation from the simplified L-M model in Eq. 2 using a single T2 map (*τ*_180_ = 12 ms) and a previously defined value of *K* (25 s^−1^). There are two unknown parameters, *T*_2*O*_ and %*SbO*_2,_ in this simplified model. As previously proposed by Wright et al., *T*_2*O*_ was first calculated for each hypoxemia stage using the reference measurement of arterial O2 saturation (measured in samples drawn by invasive catheterization) for arterial blood T2. The calculated *T*_2*O*_, predetermined *K*, and measured venous blood T2 were then used in Eq. 2 to solve for O2 saturation in the right ventricle. Approach 2: Estimate O2 saturation and other nuisance parameters by using the unconstrained optimization of the L-M model described in Eq. 1. The data consisted of multiple T2 maps, each corresponding to a different *τ*_180_ value. Approach 3: This approach was similar to Approach 2 with the exception that the values of *T*_2*O*_, *τ*_*ex*_, and *α* were learned from a training dataset. Approach 4 (our proposed approach described above): This approach was similar to Approach 2 with the exception that estimation of nuisance parameters was subjected to constraints (bounds) that were learned from a training dataset.

### Statistical analysis

All variables are reported in mean ± standard deviation. Statistical analysis was performed in MedCalc Statistical Software version 17.8 (MedCalc Software bvba, Ostend, Belgium; http://www.medcalc.org; 2017). The coefficient of variation was calculated to determine the reproducibility of blood gas measurements. Linear regression was performed to compare the relationship between the venous O2 saturation estimated using CMR against the reference venous O2 saturation measured by blood gas analysis. The systematic bias and limits of agreement between the two methods were evaluated by the Bland Altman method. A regression analysis was also performed on the differences of the two methods in the Bland Altman plots to determine any proportional bias [32]. Statistical significance was inferred for *P* < 0.05.

## Results

The measured T2 of the static phantoms with the MESE approach were 233 ms (Phantom 1) and 184 ms (Phantom 2), respectively. The quantitative T2 values measured in the four T2 maps were similar for both phantoms. The T2 values corresponding to *τ*_180_ times of 12, 15, 20, and 25 ms were 239, 239, 235, and 242 ms, respectively, for Phantom 1, and 193, 194, 192, and 196 ms, respectively, for Phantom 2.

The average baseline characteristics for all animals are listed in Table 1. The mean hematocrit fraction in all animals was 0.25 ± 0.03 (range, 0.20 to 0.29). The maximum standard deviation in hematocrit observed in any one animal was 2.5%.

**Table 1:** Baseline characteristics for animals in hypoxemia experiment

Baseline Characteristics	Mean ± SD
Heart Rate (bpm)	97 ± 18
Cardiac output (L/min)	5.4 ± 1.0
Arterial blood pH	7.45 ± 0.05
Venous blood pH	7.39 ± 0.05
Arterial pO2 (mmHg)	511 ± 18
Venous pO2 (mmHg)	45 ± 5

**Fig. 4:**
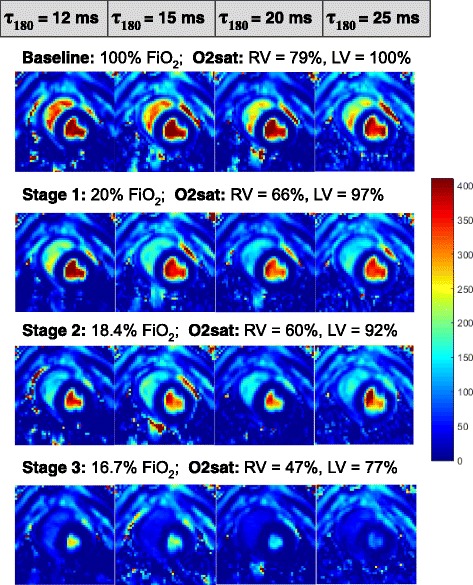
Example of blood T2 maps acquired in one animal at different stages of hypoxemia. The four T2 maps (at *τ*_180_ = 12, 15, 20, and 25 ms) acquired in an animal at four different levels of inspired oxygen (*FiO*_*2*_) are shown. The corresponding venous (RV) and arterial (LV) O2 saturation levels measured in the right and left ventricles by invasive catheterization and blood gas analysis are shown at the top of each row. Note the decrease in T2 in both arterial and venous blood pools with increasing *τ*_180_ (left to right) as well as with decreasing oxygen (top to bottom)

The heart rate and cardiac output increased with progressive hypoxemia in all animals. Arterial and venous blood T2 values decreased with lower levels of inspired oxygen. T2 of arterial and venous blood generally decreased with increasing *τ*_180._ The T2 maps acquired at four different stages of hypoxemia in one animal are shown in Fig. 4.

Thirty-three paired measurements of arterial and venous blood at different oxygen saturation levels were obtained from the seven animals. Two of the animals died during the experiment, prior to 100% O2 recovery; however, data from three O2 saturation levels could be used in one of these animals, and from one O2 saturation level in the other. Six data sets, where the venous O2 saturation fell below 40% were excluded from the analysis because rapid breathing, along with high and variable heart rates at these severe hypoxemia stages, significantly degraded image quality. Therefore, the data used in the final analysis included 27 measurements - from one hypoxemia stage in one animal, three hypoxemia stages in two animals, and five hypoxemia stages in the other four animals.

Out of these 27 measurements, two animals were randomly chosen to be used as a training set. These animals had five and three usable data sets respectively, leading to a total of eight data sets available for training. Using exhaustive search, a single optimal value, as well as a set of bounds that minimized average absolute error in estimated venous O2 saturation was chosen for each of the three nuisance parameters. These values or bounds limit the solution space and thus avoid local minima or physiologically improbable values. The remaining 19 data sets were then used for comparing the four solutions described in the methods above. The fixed or initial values and bounds applied in the NLLS curve fitting of the L-M model are listed in Table 2.

**Table 2:** Initial values and bounds applied to estimate unknown parameters of the L-M model

Estimated Parameters	Unconstrained Solution	Fixed Solution, with *T*_2*O*_, *τ*_*ex*_, and *α* values learned from training data	Constrained Solution, with *T*_2*O*_, *τ*_*ex*_, and *α* ranges learned from training data
%*SbO*_2_	0.8, [0–1]	0.8, [0–1]	0.8, [0–1]
*T*_2*O*_ (ms)	200, [0 - ∞]	350	300, [200–400]
*τ*_*ex*_ (ms)	3, [0 - ∞]	1.5	3, [0–6]
*α* (ppm)	0.5, [0 - ∞]	0.92	0.545, [0.52–0.57]

For the training set, the venous O2 saturation levels measured by catheter sampling ranged from 45% to 81%; for the testing set, the venous O2 saturation ranged from 47% to 87%. To determine the reproducibility of blood gas measurements, 14 data sets (seven each from arterial and venous blood at baseline for each animal) were evaluated. The coefficient of variation was found to be 2.6%.

For the pre-calibrated solution using the simplified L-M model and a previously defined single calibration factor *K*, the O2 saturation values estimated by CMR ranged from 57% to 88% and had an RMSE value of 8.8. O2 saturation could not be determined for one of the 19 data sets (arterial and venous O2 saturations were 100% and 87%, respectively) since the T2 value for venous blood measured slightly higher than the arterial blood T2 (283.9 ms and 274.1 ms). For the unconstrained solution to the L-M model, the O2 saturation values estimated by CMR ranged from 6% to 99% and had an RMSE value of 26.6. The mean, standard deviation, and range of the three nuisance parameters were: 6.8×10^9^ ± 2.6×10^10^ ms (165 to 1.1× 10^11^ ms) for *T*_2*O*_; 2.2×10^4^ ± 3.9×10^4^ ms (1.5 to 1.0× 10^5^ ms) for *τ*_*ex*_; and 11.37 ± 17.29 ppm (0.23 to 71.69 ppm) for *α*. For the solution with fixed, learned values for the nuisance parameters, the O2 saturation values estimated by CMR ranged from 50% to 81% and had an RMSE value of 4.5. For the proposed constrained solution, the O2 saturation values estimated by CMR ranged from 49% to 90% and had an RMSE value of 4.1. The mean, standard deviation, and range of the three nuisance parameters were: 225 ± 27 ms (200–279 ms) for *T*_2*O*_; 5.0 ± 1.1 ms (3.1–6 ms) for *τ*_*ex*_; and 0.56 ± 0.01 ppm (0.52–0.57 ppm) for *α*.

**Fig. 5:**
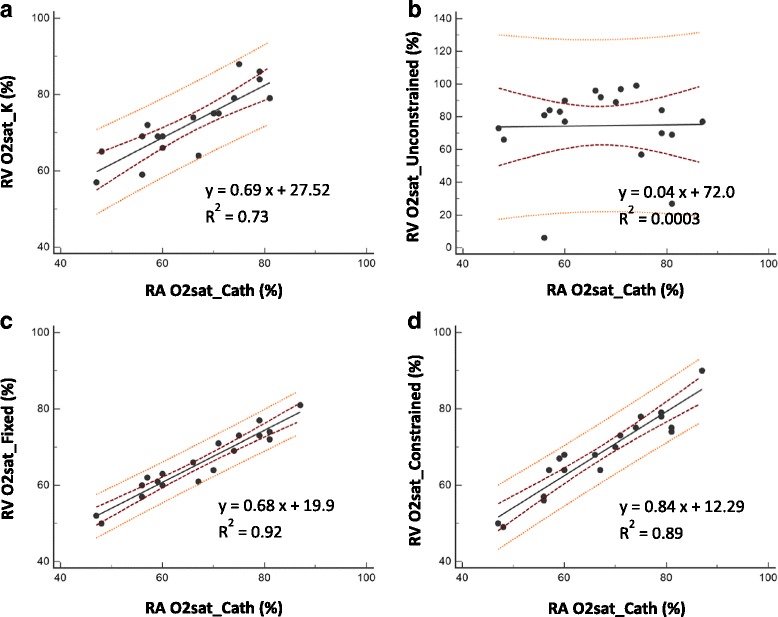
Regression plots comparing CMR measurements of O2 saturation against reference O2 saturation by catheterization. The regression plots indicate the linear relationship between CMR measures of O2 saturation (%, measured in the right ventricle) estimated from **a** global calibration, **b** unconstrained optimization, **c** fixed nuisance parameter, and **d** constrained nuisance parameter solutions to the L-M model, with the reference O2 saturation by catheter sampling (measured in the right atrium). The regression line is depicted by the solid black line. Also shown are the 95% confidence band (within the red lines) and 95% prediction band (within the orange lines). Note that the y-axis in plot (**b**) is on a different scale

**Fig. 6:**
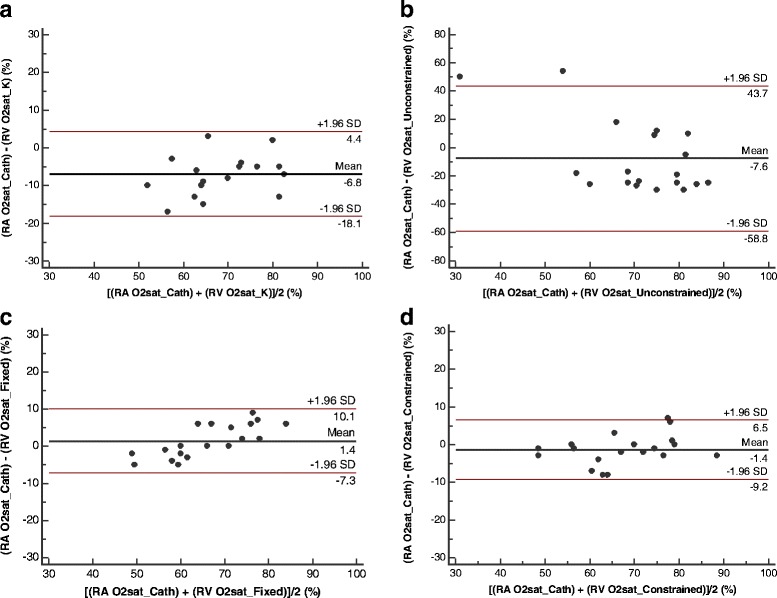
Bland Altman plots comparing CMR measurements of O2 saturation against reference O2 saturation by catheterization. The plots show the agreement between the CMR estimates of O2 saturation from the different solutions to the L-M model - **a** global calibration, **b** unconstrained optimization, **c** fixed nuisance parameter, and **d** constrained nuisance parameter - with the reference O2 saturation by catheter sampling. Note that the y-axis in plot (**b**) is on a different scale

Linear regression and Bland Altman plots comparing the different solutions for CMR estimations of O2 saturation against catheter based venous O2 saturation measurements are shown in Figs. 5 and 6, respectively. A repeated measures analysis of variance determined that the between-subjects variability was not significant (*P* = 0.49). Therefore, considering the small sample size, the different data points were treated independently in the regression analysis.

The linear relationship between blood gas and CMR based solutions to the L-M models was significant for the pre-calibrated method (y = 0.69x + 27.52, R^2^ = 0.73, *P* < 0.0001), the fixed parameter solution (y = 0.68x + 19.9, R^2^ = 0.92, P < 0.0001), and the constrained solution (y = 0.84x + 12.29, R^2^ = 0.89, P < 0.0001). The unconstrained solution did not exhibit a significant linear relationship (y = 0.04x + 72.0, R^2^ = 0.0003, *P* = 0.94). The coefficient of determination, R^2^, was the highest for the solution with the fixed nuisance parameters while the solution with the constrained parameters demonstrated the lowest offset and slope closest to 1.0.

The Bland Altman plot showed a significant systematic bias (−6.8% ± 5.7%, 95% CI = −9.7% to −4.0%, *P* = 0.001) for the solution using the pre-calibrated method. The limits of agreement ranged from −18.1% to 4.4%. The bias was the highest but non-significant for the unconstrained solution (−7.6% ± 26.2%, 95% CI = −20.2% to 5.0%, *P* = 0.22); the limits of agreement were the widest from −58.8% to 43.7%. The bias was considerably lower and non-significant for the fixed parameter solution (1.4% ± 4.4%, 95% CI = −0.7% to 3.6%, *P* = 0.18) as well as the constrained solution (−1.4% ± 4.0%, 95% CI = −3.3% to 0.6%, *P* = 0.15). The limits of agreement for the fixed parameter solution were −7.2% to 10.1% and for the constrained solution were −9.2% to 6.5%. In addition, the regression of the differences in the Bland Altman plot showed a significant slope for the unconstrained and fixed parameter solutions, indicating a significant proportional bias in O2 saturation estimated with these methods.

## Discussion

We describe a method to non-invasively determine blood oxygen saturation using quantitative T2 maps with validation against invasive blood gas analysis across a range of oxygen saturation levels in a porcine model of progressive hypoxemia. The range of venous O2 saturation examined in this study spans the wide range of normal and abnormal levels seen in cardiovascular disease. O2 saturation estimated by the proposed CMR method demonstrated a very good agreement with invasive blood gas analysis. This novel approach obviates the need for *τ*_180_-specific and patient-specific in vitro calibration, and provides an auto-calibrated estimation of venous oxygen saturation by CMR.

We exploited the dependence of blood T2 on the inter-echo spacing in the CPMG pulse train [33] to acquire a range of effective T2 values as a function of *τ*_180_ and thereby generated sufficient data to perform a reliable fit of the four unknown parameters of the L-M model. In our study, these multiple estimates of blood T2 have been acquired using single-shot, T2-prepared, SSFP quantitative T2 maps of the ventricular blood pools. The T2 maps were acquired in a free-breathing state, with the intention of designing and implementing a protocol that would be feasible in cardiovascular patients, many of whom have difficulty holding their breath.

The range of *τ*_180_ times was chosen such that a reasonable tradeoff between sensitivity to oxygen saturation and insensitivity to flow induced dephasing was achieved [5, 14, 34]. As seen in Fig. 1, the sensitivity to O2 saturation increases with increasing *τ*_180._ However, moving protons undergo greater irreversible dephasing during the longer refocusing intervals, leading to increased sensitivity to flow and associated signal loss. In the future, statistical sensitivity analysis may be used to further optimize the range and sampling of *τ*_180_.

In the interest of overall image acquisition time and practical application in the clinic, the relatively simple model described in Eq. 1 was chosen to demonstrate the proposed solution for CMR oximetry. While the same methodology presented here could be used to estimate the parameters of more complex models, an examination of the various other models that have been described in literature [21, 24, 35–37] is beyond the scope of the present work.

In the present study, four different approaches to solving the L-M model were compared; the agreement of each method with blood gas analysis is shown in Figs. 5 and 6. While unconstrained optimization clearly failed (Figs. 5b and 6b), the other three methods gave reasonable results. The use of a single global calibration factor in a simplified L-M Model (Figs. 5a and 6a), as recommended in most previous work [5, 16, 18, 19, 23], did not perform as well as the multi-parametric model. Assigning fixed values to the nuisance parameters by training on a subset of data (Fig. 5c) resulted in a stronger correlation and narrow confidence limits. It is not surprising that the fixed parameter method would perform well in these animals that exhibit greater physiological homogeneity in terms of hematocrit and nuisance variables than is likely to be encountered in a patient population. However, the proportional bias revealed by the Bland Altman plot (Fig. 6c) also indicates that this method may not be reliable across a range of O2 saturation levels. The proposed method, using subject-specific hematocrit and allowing constrained variation of the nuisance parameters, resulted in a strong correlation with slope closer to unity (Fig. 5d), and without any significant bias (Fig. 6d). These results suggest that the proposed method may translate more readily to a clinical population with widely varying physiological characteristics.

With regard to the nuisance parameters, previously reported values of *T*_2*O*_ at 1.5 T in animal, adult or pediatric blood range from 154 to 372 ms [5, 23, 25, 35, 37], which is near the fixed value of 350 ms, the range of 200 to 400 ms generated by the training data, and the calculated values of 200 to 279 ms we found. The values of *τ*_*ex*_ in the literature have been reported to vary within 0.6 to 12 ms [10, 14, 21, 33, 37–39], with more recent studies indicating values of 2 to 3 ms [35]. Our training data produced a fixed value of 1.5 ms, bounds of 0 to 6 ms, and calculated values of 3 to 6 ms; these results are closely aligned with the literature reported limits. The values for *α* in the literature, as reported for the term in Eq. 1, vary from 0.1 ppm to 1.4 ppm [14, 38]. In expanded forms of the L-M model, a comparable term to *α* is *ω*_*deo*_ − *ω*_*oxy*_, which has been reported to vary from 0.22 to 0.69 ppm [21, 35, 40]. Our training data produced a fixed *α* of 0.92, which is on the upper end of reported values, while the bounds of 0.52 to 0.57 ppm fall within reported values of *α* and *ω*_*deo*_ − *ω*_*oxy*_. Although the bounds learned from the training dataset comply with the values reported in literature, these bounds became active for six data sets (out of 19) for *T*_2*O*_, eleven sets for *α*, and nine data sets for *τ*_*ex*_ during the NLLS curve fit.

It may also be advantageous to replace the bounds with Gaussian priors for the nuisance parameters. The mean and variance of such priors could be learned via training. In contrast to bounds, Gaussian priors can accommodate extended ranges for the nuisance parameters and provide a softer mechanism to discourage divergent values due to lack of estimation sensitivity.

Besides providing non-invasive access to virtually any location in the cardiovascular system, this CMR-based method also offers the ability to assess average O2 saturation over large regions of interest. Catheter-based blood sampling in chambers where sufficient mixing has not occurred may result in inaccurate O2 saturation measurements as the catheter provides only a small, localized sample. Non-invasive measurement of O2 saturation by CMR, on the other hand, allows the spatial averaging of O2 saturation within a large region of interest within cardiac chambers or blood vessels. This provides effective “mixing” of the blood within the image plane and thus may overcome one limitation of diagnostic invasive catheterization. In the future, three-dimensional imaging techniques may be developed to provide full spatial coverage of cardiac chambers and large vessels.

### Limitations

In the absence of noise or any model mismatch, an unconstrained approach described in this paper should work reliably. In the presence of measurement noise, however, the sensitivity to estimate each of the unknown parameters is limited, which results in parameter estimation with large variance. For such cases, enforcing bounds on parameters acts as a safeguard against assigning unrealistic values to some of the nuisance parameters while still providing some room for the parameters to adapt to the data. Reliance on bounds, however, is one of the limitations of this approach because the results are partially influenced by the selection of the bounds and not entirely by the data. For the in vivo data presented, one or more nuisance parameter bounds became active (for a total of 17 out of 19 tested). Despite this limitation, our preliminary results indicate that the proposed method can outperform existing methods that use a single predetermined calibration constant.

For the hypoxemia experiment, CMR was performed in the left and right ventricles. However, we obtained venous blood samples from the right atrium to avoid any artifacts that may be caused by the catheter being positioned in the imaging plane. The right atrium is known to exhibit greater regional variability of O2 saturation within the blood pool due to blood draining from the superior and inferior vena cavae and the coronary sinus. As the blood from these vessels have different levels of oxygenation, sampling of blood from the right atrium as was done in this experiment could cause greater variability in the catheter based O2 saturation measurement; this may have degraded the correlation with the CMR-based measurement that was made in the right ventricle. In addition, the arterial and venous O2 saturation levels by blood gas analysis varied from the start to the end of imaging at some hypoxemia stages despite waiting for ten minutes for the animal to achieve a steady state. We tried to alleviate any systematic bias in the measurement of T2 by acquiring the T2 maps in a randomized order, and by taking an average O2 saturation of the blood samples obtained at the beginning and end of imaging at each hypoxemia stage as the reference standard. Despite these measures, the resulting variability could account for the small inaccuracy seen between the reference and estimated venous O2 saturation in our study.

Hyperoxia or breathing 100% oxygen can increase the amount of oxygen dissolved in plasma, which is known to increase the relaxation rates. However, a recent study by Ma et al. [41] has shown the changes in R2 to be minimal. In addition, we maintained a sufficiently long interval of five seconds between image acquisitions for all hypoxemia stages to account for any influences of T1 recovery on the T2 estimates.

Although the T2 preparation is non-selective, it is possible that high blood flow rates may result in partial inflow of blood from distal regions that have not experienced the magnetization preparation. Another possibility is that the proximity of the lungs and trachea to the vena cavae may have some influence on the uniformity of T2 preparation of blood. These conditions may have contributed to some of the regional T2 variations in the ventricles, and account for the larger variation in O2 saturation seen in some hypoxemia stages.

Because the non-invasive pulse oximeter readings obtained from the lips of the animals were found to be unreliable, invasive arterial O2 saturation was used as the reference value. With the goal of measuring O2 saturation non-invasively in patients, an invasive arterial O2 saturation measurement would not normally be available; however, in patients a finger pulse oximeter is expected to provide a reasonable reference value for arterial O2 saturation that will support the accurate estimation of venous O2 saturation.

### Future directions

CMR oximetry techniques have been previously described to estimate blood O2 saturation in pediatric and adult populations [5, 18, 23, 26]. However, these methods have not gained clinical acceptance and this may be due to the need for patient-specific in vitro calibration procedures. While other studies have performed T2 measurements across a range of *τ*_180_ [12, 13, 35], the goal of those experiments was primarily to derive predetermined calibration parameters for the estimation of O2 saturation from a single T2 measurement. However, this strategy may lead to inaccurate results in the individual patient due to the contributions from multiple patient-specific parameters besides O2 saturation to blood T2. As a simple example, a calibration factor of *K* = 25 s^−1^ applied to a venous blood sample having a T2 of 150 ms and *T*_2*O*_ of 300 ms would result in an estimated O2 saturation of 64%, assuming the calibration is performed for the normal hematocrit range. However, if the patient had anemia and the actual *Hct* was 0.25 instead of a normal value of 0.45, the true O2 saturation would be 58% if one accounted for the contribution of *Hct* to the L-M model in Eq. 1. We believe that our concept would be universally applicable for any mathematical representation of the exchange/diffusion model that describes the dependence of blood T2 to its corresponding O2 saturation as a function of the inter-echo spacing (*τ*_180_). The data could therefore, potentially be acquired with any imaging sequence that achieves T2 weighting with a train of refocusing pulses. By allowing the nuisance parameters to be estimated on a patient-specific basis, we do not have to account for the individual effects of age, gender, etc., on the hemodynamic properties of blood. With regard to applicability at a higher field strength of 3 T, where the effects of B0 and B1 inhomogeneities are greater, our preliminary experiments in a cohort of healthy volunteers [42] indicate that the method is feasible, although the validity of the method at 3 T is yet to be rigorously assessed.

Non-invasive estimation of hematocrit would render the oximetry technique truly non-invasive. As hematocrit factors in as *Hct*(1 − *Hct*) in the L-M model, a solution from T2 relaxation alone is difficult due to the possibility of multiple values being estimated. Recent studies have demonstrated the potential to estimate hematocrit and oxygen saturation from T1 measurements [43], and have also proposed a combination of T1 and T2 measurements for joint estimation of hematocrit and O2 saturation [40]. Incorporating these features may lead to a truly non-invasive oximetry technique, but is outside the scope of the current work.

The clinical application of this technique would be especially valuable in heart failure, pulmonary hypertension, and congenital heart disease patients, who may present under different hemodynamic and metabolic states, and altered blood magnetic properties than the normal population. The present study focused on an animal hypoxemia model with normal cardiovascular anatomy. Animal models of intra- and extra-cardiac shunts could also be useful to help establish the performance of the technique under conditions that may be found in a congenital heart disease population.

## Conclusion

A novel CMR method for patient-specific, non-invasive estimation of blood O2 saturation in the heart was implemented and validated across a range of physiological and pathological O2 saturation levels. In this preliminary evaluation that primarily sought to establish proof of concept, effective T2 measurements of arterial and venous blood using T2-prepared bSSFP T2 maps were acquired at distinct inter-echo spacings and fit to the L-M model to non-invasively estimate O2 saturation along with three nuisance parameters. The estimation of venous O2 saturation from these effective T2 measurements of the blood pool was in good agreement with the reference measurement obtained by invasive catheterization.

## Abbreviations

bSSFP: balanced steady state free precession
CMR: cardiovascular magnetic resonance
CPMG: Carr-Purcell Meiboom Gill
Hct: hematocrit
L-M: Luz-Meiboom
MESE: multi-echo turbo spin echo
MLEV: Malcolm Levitt
MR: magnetic resonance
NLLS: non-linear least squares
O2: oxygen
RBC: red blood cells
